# Loss and psychosocial factors as determinants of quality of life in a cohort of earthquake survivors

**DOI:** 10.1186/s12955-015-0209-5

**Published:** 2015-02-06

**Authors:** Vahe Khachadourian, Haroutune K Armenian, Anahit Demirchyan, Armen Goenjian

**Affiliations:** School of Public Health, American University of Armenia, Yerevan, Armenia; Department of Epidemiology, Fielding School of Public Health, UCLA, Los Angeles, CA USA; Department of Psychiatry and Biobehavioral Sciences, UCLA/Duke University National Center for Child Traumatic Stress, UCLA, Los Angeles, CA USA

**Keywords:** Earthquake, Quality of life, Psychopathology, Material loss, Social support

## Abstract

**Background:**

Despite the existing evidence of a long lasting effect of disaster related experiences on physical and psychological health, few studies have evaluated long-term quality of life (QOL) outcomes of disaster survivors and the factors associated with such outcomes.

**Methods:**

23 years after the 1988 Spitak earthquake in Armenia, the associations of demographic characteristics, trauma exposure and psychosocial variables on QOL were explored among a cohort of 725 exposed individuals. The EQ-5D-5 L instrument was applied to measure QOL of participants. Multivariate linear and ordinal logistic regressions were applied to evaluate the determinants of QOL and its underlying five domains (mobility, self-care, usual activity, pain/discomfort, and anxiety/depression).

**Results:**

Older age, current depression, post-traumatic stress disorder and anxiety symptoms were negatively associated with QOL. Additionally, those with severe losses (who did not receive any financial/material aid) had significantly poorer QOL outcomes, with higher odds of mobility difficulties (OR = 1.86, p < 0.05), self-care difficulties (OR = 2.85, p < 0.05), and mood problems (OR = 2.69, p < 0.05). However, those with severe earthquake related losses who received financial/material aid reported less self-care difficulties (OR = 0.21, p < 0.05) usual activity difficulties (OR = 0.40, p < 0.05), and mood problems (OR = 0.44, p < 0.05). Finally, each unit increase in current social support score was found to be significantly associated with a better QOL outcome and better self-reported outcomes across all underlying domains of QOL.

**Conclusions:**

These findings suggest that earthquake related loss and concurrent psychopathology symptoms can have adverse impact on the QOL of survivors. They also indicate that well-targeted post-disaster financial/material aid and social support should be considered as means for improving the long-term QOL outcomes of disaster survivors.

What is already known on this subject?

- Research have shown that disasters have adverse impacts on short-term physical and mental health of survivors, negatively affecting their quality of life.
- However, studies evaluating long-term impact of disasters on quality of life of survivors are limited.

What this study adds?

- The current study followed earthquake survivors over two decades and found that the impact of disaster related loss on QOL can extend to the third decade after the disaster.
- The findings suggested that intervening variables such as concurrent physical illnesses and mental illnesses are negatively associated with QOL measures after two decades.
- Post-earthquake financial aid to those with severe loss was positively associated with QOL even two decades after the disaster.

## Background

Health consequences of natural disasters, particularly earthquakes, are a major public health concern. There is a large body of evidence on the short-term as well as growing evidence on the long-term mental and physical health impact of earthquakes [1-4]. However, few studies have explored the effects of post-earthquake physical and mental health consequences and other earthquake-related experiences on the Quality of Life (QOL) of survivors. Most of the studies that have evaluated the QOL found a negative association between post-disaster psychopathological outcomes and QOL [5,6]. Tsai et al. [6] found that the psychological response to the Chi-Chi earthquake and its sequelae of post-traumatic stress disorder (PTSD) have a significant negative association with QOL. According to their findings, groups with persistent PTSD symptoms from six months to three years post-earthquake had the poorest QOL, while those who suffered from PTSD at six months but recovered after three years had better QOL. PTSD is not the only psychopathology found to be associated with QOL. Among a sample of adolescents exposed to the Parthina earthquake in Greece, Goenjian et al. [7] reported that at 32 months post-earthquake, in addition to the negative impact of PTSD, individuals with higher depressive symptoms also had on average lower QOL scores.

Earthquake-related experiences may also influence QOL in the long-term. Few studies have explored the associations between earthquake related material loss, level of post-earthquake social support and the QOL and found that social support was positively associated with QOL measures [5,8,9]. In contrast, earthquake related material losses and higher severity of exposure to trauma were associated with lower QOL scores among survivors [5,8].

Our own studies from the 1988 earthquake in Armenia have shown that earthquake exposure, related material loss, and social and financial support were associated with post-earthquake-psychopathology [10,11]. Other researchers have also found that post-earthquake-psychopathology was associated with QOL [5,8]. Nonetheless, only a few studies have evaluated the potential longer-term (more than 5 years) association between earthquake exposure and QOL. The longest-term study to date reported by Ceyhan et al. [12] noted a lower QOL among earthquake-exposed students compared to unexposed students six years after the Marmara earthquake.

On December 7, 1988, the Spitak earthquake, with a magnitude of 6.9 on the Richter scale, struck the northern part of Armenia, resulting in extreme destruction, homelessness (500,000), serious injuries (100,000), and deaths (25,000). Our study aimed to explore the associations between earthquake-exposure experiences, psychopathology and socio-demographic characteristics of survivors, and long-term QOL measures, 23 years after the Spitak earthquake.

## Methods

### The study population

The study population consisted of the employees of the Ministry of Health (MOH) of the Soviet Republic of Armenia living in the earthquake region and their family members. Their data were identified from the national payroll office. A total of 32,743 individuals were located, assented to participate and were included in the baseline cohort of the study (phase I) (78% of the total sample of potential study participants). After the completion of phase I, a geographically stratified sub-sample of the original baseline cohort, consisting of 1773 adults, was selected to be involved in the three follow-up phases conducted from 1991 to 2012 (phase II in 1991, phase III during the 1992–1994 period, and phase IV in 2012). This stratified sub-sample included a higher proportion of individuals from the severely impaired areas to better assess the association of exposure with the outcomes of interest.

Up to phase III, the follow-up information was successfully obtained for 97% of the initial population [2]. For the 23-year follow-up (phase IV), 1487 (84%) of the original sample of 1773 were located, and 286 (16%) could not be located. Out of those located, 309 (21%) were dead, 300 (20%) had permanently moved out of country, 89 (6%) were not able to participate (mainly because of severe health conditions or temporarily not being available), 725 (49%) assented to participate and 64 (4%) refused. In 2012, we were able to obtain follow-up information for 1423 (80%) of the initial 1773 sample. Table 1 compares selected characteristics of phase IV participants 725 with those of all non-participants including those who were not located, those who refused, those who were dead and those who left the country.

**Table 1:** **Descriptive statistics of quality of life outcome and other variables of interest among the cohort of the 1988 Armenian earthquake survivors**

**Characteristic**	**Participants in 2012**	**Non-participants in 2012**
	**n = 725**	**n = 1048**
Age in 2012^a^, mean (SD)	58.95 (12.07)	63.08 (15.83)
Female gender^a^, %	67.9%	50.3%
Length of education (year)^a^, mean (SD)	12.18 (2.42)	11.37 (3.22)
Earthquake related material loss		
none to minimum loss, %	60.6%	57.4%
moderate loss, %	22.9%	24.0%
severe loss, %	16.6%	18.5%
Post-earthquake financial/material aid, %	35.2%	36.0%
Earthquake related death in the family, %	9.5%	9.0%
Earthquake related injury, %	8.7%	7.9%
PTSD in 1991^b^, %	49.0%	50.0%
Depression in 1991^b^, %	51.5%	51.7%
PTSD in 2012^b^, %	13.0%	-
Depression in 2012^b^, %	26.4%	-
Anxiety in 2012^b^, %	28.9%	-
Alcohol abuse in 2012, %	4.3%	-
Current smoker in 2012, %	18.5%	-
Social support score in 2012^c^, mean (SD)	17.90 (3.77)	-
Number of physical illnesses in 2012^d^, mean (SD)	2.80 (1.97)	-
Quality of life score (health index), mean (SD)	0.68 (0.25)	-

^a^Significant difference (p-value <0.05).

^b^Binary variable created using a cut-off level.

^c^Social support score range was 0–28.

^d^Number of physical illnesses range was 0–15.

SD: standard deviation.

### Measurements

#### Socio-demographic characteristics, earthquake exposure and related experiences

A wide range of previously (1990–1992) collected socio-demographic characteristics, including age, gender, and education, was updated during phase IV (2012). Additionally, during the same phase, information on self-reported socio-economic status (SES) of participants was obtained.

Data on earthquake exposure, including earthquake-related injuries of respondents and deaths among respondent’s family members, were collected during phase I (1990–1992) and verified during phase III (1992–1994). During phase I (1990–1992), in addition to the information on the earthquake related loss (including loss of home, finance, car, furniture and everyday life appliances), data was obtained on the financial/material aid received post-earthquake. Earthquake related material loss was divided into three levels of none to minimum loss, moderate loss, and severe loss. Exposures to life time stressful and traumatic events, including natural disasters, violence, life threatening accidents, sudden unexpected death of a loved one, participation in war, and any other horrifying or life threatening event were collected during phase IV (2012). The number of exposures were summed into a continuous variable (range 1–7). Data on participant’s smoking and drinking habits were obtained in phase IV (2012). Drinking two or more portions of alcohol on four or more days per week was considered as alcohol abuse.

#### Physical health

During phase IV (2012), detailed self-report of physical health was obtained. The health questionnaire included current cardiovascular diseases, respiratory diseases, diabetes, arthritis, migraine, allergic diseases, and digestive tract diseases. The total number of physical illnesses was used for analyses (range 0–15).

#### Mental health

During phase II (1991) of this study, the participants underwent screening for depression, anxiety, and PTSD symptoms, using instruments based on the DSM-III-R diagnostic criteria [13]. Elaboration on the instruments and the cut-off levels used is described elsewhere [10,11]. During phase IV, subjects were assessed for the same psychological conditions. The Post-traumatic checklist-civilian version (PCL-C) was used to assess PTSD symptom severity. [14,15]. This instrument is internationally recognized and widely used. The PCL-C is a 17-item scale, which has a 5-level ordinal response options from *not at all* to *extremely* (1–5) with a summative score ranging from 17 to 85. A cut-off of 52 or above on the PCL-C was used to distinguish possible PTSD cases from non-cases. Anxiety symptoms were assessed by using the 10 item Symptom Checklist 90-Revised [16]. This scale has a 5-point response scale and its summative score ranging from 0 to 40 was used to assess the symptoms of anxiety, and anyone scoring above 10 on this scale was considered as possibly anxious. Depression symptoms were measured via the revised/adapted [17] Center for Epidemiological Studies-Depression scale (CES-D) [18] consisting of 16 negatively-worded items with a 4-level response scale presenting the frequency of symptoms from less than one day per week to 5–7 days per week (total score ranging from 0 to 48). A cut-off score of 13 or above out of 48 was used as indicative for possible depression [18].

Self-perceived family/friend related social support score ranging from 0 to 28 (with higher scores reflecting better social support) was generated from the responses to seven questions administered at phase IV (2012).

#### Quality of life

The official Armenian translation of the EQ-5d-5 L was used to assess QOL [19]. This is a short instrument consisting of five questions which target *mobility*, *self-care*, *usual activity*, *pain/discomfort* and *anxiety/depression*. Each question had a 5-level ordinal response scale indicating the level/frequency of difficulties experienced in each domain. The instrument provides a health index (a population specific-weighted index) [20], which is a valuable measure for assessing economic burden associated with different conditions and can also be used for evaluating the cost effectiveness of various interventions addressing QOL [19].

### Ethical consideration

The study protocols for the phases I, II and III were approved by the Institutional Review Board (IRB) of Johns Hopkins School of Hygiene and Public Health, and for the phase IV by the IRB of the American University of Armenia. During all phases, the interviews were conducted only after describing the study, its objectives, potential risks and benefits and after obtaining informed oral consent to participate.

### Data analysis

We used STATA 12.0 to conduct statistical analyses. For each scale - depression (CES-D), anxiety (SCL-90-R) and PTSD (PCL-C), cases with three or less missing values on a scale were maintained in the analysis with the missing values replaced by average value of answered items on that scale, while the cases with more than three missing values on a scale were excluded from the analysis.

We employed a bivariate linear regression analysis to investigate the association between the QOL score (health index) and psychosocial factors, as well as earthquake related experiences, including earthquake related material loss and post-earthquake support. We applied multivariate linear regression analysis to evaluate the true association of variables of interest with QOL while controlling for potentially confounding factors, including all variables with a p-value of less than 0.25 in the bivariate analyses. We initially considered an interaction term between post-earthquake financial/material aid (two-level) and earthquake related material loss (three-level), however, as the effect of material loss among those with none to minimum loss and moderate loss were statistically similar, they were collapsed into a single category resulting into a two-level material loss variable. Finally, after assuring that the data met the parallel-lines model assumptions, ordinal regression was applied to evaluate the association between variables of interest (psychosocial factors as well as earthquake related experiences including earthquake related material loss and post-earthquake support) and the five domains of QOL instrument: *mobility*, *self-care*, *usual activity*, *pain/discomfort*, and *anxiety/depression*.

## Results

Table 1 presents descriptive data on demographic characteristics, social support, earthquake related material loss, post-earthquake financial/material aid, earthquake related death in the family, earthquake related injury, prevalence of PTSD and depression symptoms for the years 1991 and 2012, the QOL (health index) of phase IV participants, as well as some baseline characteristics of all non-participants.

Table 2 presents the results of bivariate linear regression analyses of the relation between the QOL score and each of the variables listed in Table 1. Concurrent psychopathology, including possible anxiety, depression and PTSD were negatively associated with QOL scores, while depression and PTSD in 1991 were not significantly associated with QOL scores. Other items demonstrating significant negative associations with QOL included age, female gender, earthquake related material loss, number of stressful life events and number of reported physical illnesses. Social support score was positively associated with QOL scores.

**Table 2:** **Bivariate linear regression analysis of quality of life scores (health index) of the 1988 Spitak earthquake survivors**

**Variables**	**Bivariate linear regression**	
	**Coefficient**	***p-value***
Age (years)	−0.007	<0.001
Female gender	−0.054	0.007
Length of education (years)	0.019	<0.001
Socio-economic status in 2012^a^	0.016	<0.001
Earthquake related material loss^b^	−0.013	0.016
Post-earthquake financial/material aid	−0.026	0.183
Post-earthquake financial/material aid to those with severe material loss	0.077	0.065
Social support score^c^	0.025	<0.001
Earthquake related death in the family	−0.014	0.662
Earthquake related injury	−0.016	0.642
Number of life time stressful events	−0.024	<0.001
Number of physical illnesses in 2012^d^	−0.056	<0.001
PTSD in 1991^e^	−0.026	0.163
Depression in1991^e^	−0.019	0.315
PTSD in 2012^e^	−0.233	<0.001
Depression in 2012^e^	−0.258	<0.001
Anxiety in 2012^e^	−0.208	<0.001
Alcohol abuse in 2012	0.052	<0.001
Current smoker in 2012	0.068	0.005

^a^Socioeconomic status: 0 = low, 1 = average, 2 = high.

^b^Earthquake related material loss was reported in phase I (1990–1992). Material loss variable was divided into three levels: none to minimum loss = 0, moderate loss = 1, and severe loss = 2.

^c^Social support score range was 0–28.

^d^Number of physical illnesses range was 0–15.

^e^Having a score equal/above the selected cut-off level.

Table 3 shows the results of the multivariate linear regression analysis. As indicated, baseline psychopathology (phase II) had no statistically significant independent relationship with current QOL. The concurrent psychopathology symptoms, including depression, anxiety and PTSD symptoms, were among the factors negatively associated with QOL. Although in bivariate linear regression results the negative coefficients of depression, anxiety and PTSD symptoms were similar, multivariate linear regression showed that the coefficient for depression symptoms was greater than for the others. The results also indicated that every additional physical illness was associated with 0.033 reduction of QOL score (health index). Age and earthquake related material loss were also negatively associated with QOL scores, while social support and financial/material aid to those with severe earthquake related material losses were positively associated with QOL score.

**Table 3:** **Multivariate linear regression model of quality of life scores (health index) of the 1988 Spitak earthquake survivors**

	**Multivariate linear regression**			
**Variables**	**Coefficient**	**95%** **CI**		***p-value***
Age (years)	−0.005	−0.006	−0.004	<0.001
Gender	−0.019	−0.062	0.004	0.387
Male = 0				
Female = 1				
Length of education (years)	0.004	−0.002	0.010	0.165
Socio-economic status in 2012	0.013	−0.006	0.032	0.186
low = 0				
average = 1				
high = 2				
Earthquake related material loss	−0.038	−0.063	−0.014	0.002
none to minimum = 0				
moderate = 1				
Severe = 2				
Post-earthquake financial/material aid	−0.023	−0.055	0.010	0.175
no = 0				
yes = 1				
Post-earthquake financial/material aid to those with severe material loss	0.075	0.009	0.141	0.025
no = 0				
yes = 1				
Social support score (0–28)	0.009	0.004	0.013	<0.001
Number of physical illnesses in 2012 (0–15)	−0.033	−0.041	−0.025	<0.001
PTSD in1991	0.010	−0.020	0.040	0.513
no = 0				
yes = 1				
PTSD in 2012	−0.072	−0.109	−0.036	0.002
no = 0				
yes = 1				
Depression in 2012	−0.108	−0.147	−0.069	<0.001
no = 0				
yes = 1				
Anxiety in 2012	−0.072	−0.109	−0.036	<0.001
no = 0				
yes = 1				
Alcohol abuse in 2012	0.049	−0.024	0.121	0.189
no = 0				
yes = 1				
Smoking in 2012	0.000	−0.051	0.052	0.992
no = 0				
yes = 1				

For the entire sample, post-earthquake financial and material help was not associated with QOL. However, there was a statistically significant positive association between post-earthquake financial/material aid and QOL among those individuals with severe earthquake related material loss (Table 3).

Table 4 shows the results of the multivariate ordinal regression analysis for each of the five domains of the QOL instrument. The adverse effect of current psychopathology was not limited to the *anxiety/depression* domain, but expanded over the other domains. For instance, having PTSD symptoms increased the odds of reporting difficulties in *self-care* (1.83), and *usual activity* (2.36) domains. Likewise, depression was significantly associated with the same two domains, and additionally with *pain/discomfort*. Those with anxiety symptoms had two times higher odds of experiencing *pain/discomfort* as compared to those without anxiety. The higher number of physical illnesses was also associated with worse outcomes across all domains. The negative impact of severe earthquake related material loss was significant for the *mobility*, *self-care*, and *anxiety/depression* domains. Although financial/material aid was not positively associated with any of the QOL domains, a statistically significant interaction term was found; those with severe earthquake related material loss who received financial/material aid had statistically significantly better outcome in *self-care, usual activity*, and *anxiety/depression* domains. Social support was found to be a statistically significant protective factor across the five domains of QOL.

**Table 4:** **Ordinal logistic regression model on determinants of difficulties reported on each of five subscales (domains) of quality of life among 1988 Spitak earthquake survivors**

	**Subscales (domains) of QOL**				
	**Odds Ratio of reporting worse on the QOL’s domains**				
**Variables**	**Mobility**	**Self-care**	**Usual activity**	**Pain/Discomfort**	**Anxiety/Depression**
Age in 2012 (years)	1.07*	1.08*	1.05*	1.03*	1.01
Earthquake related material loss					
None to minimum	1.00	1.00	1.00	1.00	1.00
Moderate	1.57*	1.67*	1.53*	1.09	1.62*
Severe	1.86*	2.85*	1.49	1.69¶	2.69*
Post-earthquake. financial/material aid	1.19	1.46	1.21	1.09	1.07
Post-earthquake. financial/material aid among those with severe material loss	0.67	0.21*	0.40*	0.86	0.44*
Social support score	0.91*	0.94*	0.91*	0.96*	0.93*
Number of physical illnesses	1.30*	1.25*	1.26*	1.38*	1.24*
PTSD in 2012^a^	1.22	1.83*	2.36*	1.24	2.04*
Depression in 2012^a^	1.42¶	1.68*	1.73*	1.72*	3.46*
Anxiety in 2012^a^	1.55*	1.22	1.03	2.06*	3.35*

Note: In addition to the variables included in the table, we controlled for all variables which had a p-value <0.25 in the Table 3; including alcohol abuse in 2012, length of education, and socio-economic status in 2012 (all these variables were insignificant in the model).

*p-value <0.05.

^¶^p-value <0.10.

^a^Having a score equal/above the selected cut-off level.

## Discussion

To our knowledge, this is the first epidemiologic follow-up study that has measured the association between socio-demographic, earthquake exposure, physical health, and psychopathological variables and QOL among survivors two decades after a major disaster. The data collection was done in four different study phases, and for some important factors data was repeatedly collected in various study phases, which minimized potential recall biases and reporting errors. Despite the fact that two decades had passed since the baseline study phase, we were able to track 80% of the initial group of 1773.

The study had some limitations. The collected data were all self-reported, which may be subject to recall bias, nevertheless, considering the lack of a comprehensive medical records and the limited resources, this was the only feasible method to use. However, considering that the baseline population was made of Ministry of Health employees including a large proportion of health professionals with better access to health services, we expected better self-reporting of morbidity and medical outcome data than the general population in our cohort. Another limitation is the lack of QOL measures in earlier study phases. Such data would have given us the opportunity to study the association of earthquake related factors with the change of QOL over time.

The multivariate regression analyses indicated that symptoms of concurrent psychopathology were negatively associated with QOL (even though symptoms of baseline psychopathology measured in phase II were not independently associated with QOL). The associations of current depression and anxiety with QOL were not limited to the *anxiety/depression* domain. Those with current depression had significantly higher odds of having poor outcomes in all domains of QOL. Current anxiety was also related to higher rates of experiencing *pain/discomfort* and difficulties with *mobility*. These results are consistent with findings of other studies (including a meta-analysis), showing adverse effects of anxiety, depression and other mental conditions on QOL [21-23]. The impact of depression and anxiety on *mobility*, *usual activity*, and *pain/discomfort* may lead to increased utilization of healthcare services and decreased job performance and productivity. Such problems can have a negative impact on social activities and family relationships.

Consistent with other research [7,24-26], current PTSD symptom severity was associated with a lower QOL in this study population. Those with a PTSD score above 51 on the PCL-C (cut-off for estimated diagnosis of PTSD) had poorer outcomes in the *anxiety/depression* domain. This is consistent with prior findings indicating a strong correlation between PTSD, depression, and anxiety [27,28]. PTSD and possible comorbid psychiatric disorders could contribute to poorer outcomes observed in *anxiety/depression* domain. Those with PTSD symptoms above the cutoff level applied also reported difficulties in performing usual activities, which is in line with both DSM-IV criteria for PTSD [29] and other studies [30,31] showing that PTSD is associated with various types of functional impairments.

The cross sectional measurements of current psychopathology symptoms and QOL outcomes limit us in making conclusions regarding the direction of such associations. The association of psychopathology with QOL scores may be a bidirectional relation whereby QOL and mental health are modified by the effect on one another. A similar alternative causation between health and SES has been previously described by Adler and colleagues [32]. Longitudinal studies of these measures would clarify the direction of the relation between them. Another possibility of the association is that both psychopathology and QOL share a common pathway of causality.

The lack of association between baseline psychopathology (phase II) and QOL and the existence of an association between concurrent psychopathology and QOL suggests that intervening variables rather than psychopathology that occurred decades earlier impacts current QOL. For example, the number of physical illnesses was one of the possible intervening variables strongly associated with all domains of QOL.

Even two decades after the disaster, the earthquake related material losses were negatively associated with survivors’ current QOL. Earlier studies, based on phases I and II data, have shown that those with severe earthquake related material loss had higher rates of depression and PTSD, and that post-earthquake financial support did not show an immediate impact on psychopathology [2,11]. However, our data indicated that the impact of earthquake related material loss on QOL was independent of its indirect adverse effect, mediated by higher rates of physical and mental morbidity, and the association of amount of material losses to QOL remained significant even after controlling for physical illnesses and past/concurrent psychopathology. Although there are studies which have reported a negative association between earthquake related material losses and QOL [5,8], the present study extends the relevance of the association to over two decades.

Even though post-earthquake financial/material aid had no effect on the QOL among the whole sample, it had significant positive effect on overall QOL and specifically on its *self-care*, *usual activity*, and *anxiety/depression domains* among those who experienced severe earthquake related material loss compared to those who had similar level of loss but did not receive any aid and support. These findings suggest that financial/material aid should be prioritized to those who have suffered serious losses. And those with serious losses have to be prioritized.

Social support was another factor positively associated with overall QOL and all the domains of it. The observed positive effect of social support on QOL extends findings of shorter term studies conducted among earthquake survivors in China and Taiwan [6,8,33].

This study highlights the negative impact of mental and physical morbidity on QOL. More importantly, the findings demonstrate the necessity of close monitoring of earthquake survivors, specifically those with severe earthquake related material losses. The present findings indicate that financial/material aid provided soon after a disaster to those with severe material losses would be beneficial intervention with regard to their QOL. Finally, provision of social support and enhancement of existing social networks may benefit the QOL of survivors.
